# *Ribes fasciculatum* Leaf Water Extract Enhances Macrophage Immune Responses via TLR4-Mediated JNK and NF-κB Activation in RAW264.7 Cells

**DOI:** 10.4014/jmb.2607.07022

**Published:** 2026-09-04

**Authors:** So Jung Park, Jeong Won Choi, Hyeok Jin Choi, Gyeong Eun Im, Bo Hee Seo, Ha Eun Kim, Kwan Been Park, Seon A Kim, Jenna Jung, Myung Suk Choi, Jin Boo Jeong

**Affiliations:** 1 Department of Forest Sciences, Gyeongkuk National University, Andong 36729, Republic of Korea; 2 Department of Forest Resources, Gyeongsang National University, Jinju 52828, Republic of Korea; 3 Department of Environmental Forest Science & Institute of Agriculture and Life Science, Gyeongsang National University, Jinju 52828, Republic of Korea

**Keywords:** *Ribes fasciculatum*, Immunostimulation, Macrophage activation

## Abstract

This study investigated the immunostimulatory activity and underlying mechanism of *Ribes fasciculatum* leaf water extract in RAW264.7 macrophages. Among extracts prepared using water or ethanol-containing solvents, only the water extract induced NO production. Further evaluation of extraction temperature and time identified the extract prepared at 20°C for 24 h (RFL-DW20) as the most active sample under the tested conditions, with NO-inducing activity increasing after 12 h of extraction and reaching a plateau by 24 h. RFL-DW20 increased NO and PGE2 production, upregulated iNOS and COX-2 expression, and enhanced IL-6 and TNF-α production without cytotoxicity. RFL-DW20 also increased IL-1β mRNA expression but did not significantly alter extracellular IL-1β levels. RFL-DW20 also increased neutral red uptake, indicating enhanced macrophage functional activity. The immunostimulatory effects of RFL-DW20 were strongly inhibited by TAK-242, SP600125, and BAY 11-7082, suggesting the involvement of TLR4, JNK, and NF-κB signaling. RFL-DW20 activated MAPK and NF-κB signaling, including rapid IκBα phosphorylation/degradation and p65 phosphorylation, while pharmacological inhibition identified JNK and NF-κB as major functional mediators of the NO response. TAK-242 markedly suppressed RFL-DW20-induced JNK and p65 phosphorylation. Ethanol precipitation further enriched its NO-inducing activity. These findings suggest that RFL-DW20 activates macrophages through the TLR4/JNK/NF-κB signaling axis and may serve as a natural immune-stimulating material.

## Introduction

The immune system is an essential host defense network that protects the body against invading pathogens, abnormal cells, and harmful endogenous or exogenous stimuli [1]. However, immune competence can be compromised by aging, chronic stress, malnutrition, disease conditions, and other environmental or physiological factors, resulting in increased susceptibility to infection and reduced responsiveness to immune challenges [2]. In particular, immunosenescence is characterized by functional deterioration of both innate and adaptive immunity and is closely associated with poor vaccine responses, increased infection risk, and higher incidence of age-related diseases [3]. Therefore, strategies that appropriately reinforce host immune defense without causing excessive or uncontrolled inflammation are of considerable interest for the development of functional foods, nutraceuticals, and immune-supporting natural products [4].

Macrophages are central effector cells of innate immunity and play a pivotal role in the initiation, amplification, and coordination of immune responses [5, 6]. They recognize danger signals through pattern-recognition receptors, engulf foreign particles by phagocytosis, produce immune mediators, and present antigens to lymphocytes, thereby linking innate and adaptive immune responses [5, 6]. Activation of macrophages can enhance host defense by increasing phagocytic capacity and promoting the production of immune-regulatory mediators, including nitric oxide (NO), prostaglandin E2 (PGE2), and cytokines such as interleukin-6 (IL-6) and tumor necrosis factor-α (TNF-α) [7-9]. NO generated by inducible nitric oxide synthase (iNOS) contributes to macrophage-mediated antimicrobial and cytotoxic defense mechanisms, whereas cyclooxygenase-2 (COX-2)-derived PGE2 and pro-inflammatory cytokines participate in the regulation of immune-cell communication and inflammatory responses [7-9]. Thus, macrophage activation, when properly regulated, represents an important cellular event for improving innate immune responsiveness [10]. Toll-like receptors (TLRs) are major pattern-recognition receptors that detect conserved microbial or endogenous danger-associated molecular patterns and initiate innate immune signaling [11]. Among them, TLR4 is one of the best-characterized receptors involved in macrophage activation. Engagement of TLR4 activates downstream signaling cascades, including mitogen-activated protein kinases (MAPKs) and nuclear factor-κB (NF-κB), which regulate the transcription of genes encoding iNOS, COX-2, IL-6, TNF-α, and other immune mediators [11, 12]. NF-κB is a central transcriptional regulator of immune and inflammatory responses, while MAPK subfamilies, including c-Jun N-terminal kinase (JNK), contribute to the fine control of macrophage activation and cytokine production [11, 13]. Accordingly, identifying natural substances that activate macrophage immune responses through defined TLR4-dependent signaling pathways is important for the mechanistic evaluation of candidate immune-enhancing materials.

Natural products have long been investigated as potential sources of immunomodulatory agents because of their structural diversity, biological compatibility, and suitability for dietary or functional applications [14]. In particular, plant-derived polysaccharides and water-soluble macromolecules have received increasing attention as biological response modifiers that can activate macrophages by enhancing NO production, cytokine secretion, and phagocytic activity through receptors such as TLR4, TLR2, dectin-1, complement receptor 3, mannose receptor, and scavenger receptors [15]. Previous studies have shown that several plant polysaccharides stimulate RAW264.7 macrophages through TLR4-mediated NF-κB and MAPK signaling, supporting the potential of plant-derived water extracts as immune-enhancing materials [16, 17]. Nevertheless, the immunostimulatory efficacy and molecular mechanisms of many forest plant resources remain insufficiently characterized. *Ribes fasciculatum* (*R. fasciculatum*), commonly known as Korean crowberry currant, is a deciduous shrub distributed in East Asia and has been traditionally used in Korea as a medicinal plant resource [18]. Previous studies have reported biological activities of *R. fasciculatum*, including anti-obesity [19, 20], antioxidant [21], anti-aging [21], and allergy-relief effects [18]. However, despite these reported pharmacological properties, the immunostimulatory potential of *R. fasciculatum* leaves and the underlying macrophage-activating mechanisms have not been clearly elucidated.

In the present study, we investigated the immunostimulatory activity of *R. fasciculatum* leaf water extract (RFL-DW20) in RAW264.7 macrophages and explored its underlying mechanism of action. We evaluated the effects of RFL-DW20 on NO and PGE2 production, iNOS and COX-2 expression, cytokine responses including IL-6, TNF-α, and IL-1β, and neutral red uptake as an indicator of macrophage phagocytic activity. Furthermore, we examined whether RFL-DW20-induced macrophage activation is mediated through TLR4-dependent JNK and NF-κB signaling. Our findings provide mechanistic evidence that RFL-DW20 enhances macrophage immune responses via activation of the TLR4/JNK/NF-κB signaling axis, suggesting its potential as a natural immune-stimulating functional material.

## Materials and Methods

### Chemical Reagents

Lipopolysaccharide (LPS, cat. no. L2630), neutral red (cat. no. N7005), 3-(4,5-dimethylthiazol-2-yl)-2,5-diphenyltetrazolium bromide (MTT, cat. no. 475989), and Griess reagent (cat. no. G4410) were obtained from Sigma-Aldrich (USA). Chemical inhibitors such as polymyxin B (PMB, LPS-neutralizing agent, cat. no. P1189), laminarin (Dectin-1-blocking ligand, cat. no. L9634,), mannan (mannose receptor-blocking ligand, cat. no. M7504), TAK-242 (TLR4, inhibitor, cat. no. 614316), C29 (TLR2 inhibitor, cat. no. SML3817), PD98059 (cat. no. P215), SB203580 (cat. no. 559398), SP600125 (cat. no. S5567), and BAY 11-7082 (cat. no. 196870) were purchased from Sigma-Aldrich. Antibodies against inducible nitric oxide synthase (iNOS, cat. no. 2982), cyclooxygenase-2 (COX-2, cat. no. 12282), phospho-ERK1/2 (p-ERK1/2, cat. no. 4377), total ERK1/2 (t-ERK1/2, cat. no. 9102), phospho-p38 (p-p38, cat. no. 9211), total p38 (t-p38, cat. no. 9212), phospho-JNK (p-JNK, cat. no. 9251), total JNK (t-JNK, cat. no. 9258), phospho-IκBα (p-IκBα, cat. no. 2859), IκBα (cat. no. 4814), phospho-NF-κB p65 (p-p65, cat. no. 3033), total NF-κB p65 (t-p65, cat. no: 8242), and β-actin (cat. no. 5125) were purchased from Cell Signaling Technology (USA). HRP-conjugated anti-rabbit IgG (cat. no. 7074) and HRP-conjugated anti-mouse IgG (cat. no. 7076) were used as secondary antibodies.

### Sample Preparation

Leaves of *R. fasciculatum* were collected from three locations in Gyeongsangnam-do, Republic of Korea, in April 2026: Sancheong-gun (35°17′07.39″N, 127°51′36.93″E), Jinju-si (35°11′00.55″N, 128°14′29.08″E), and Changwon-si (35°15′05.86″N, 128°33′35.81″E). The plant materials were taxonomically authenticated by Professor Myung Suk Choi at Gyeongsang National University. Voucher specimens corresponding to the materials collected from Sancheong-gun, Jinju-si, and Changwon-si were deposited at the Korea National Arboretum Herbarium (Republic of Korea) under voucher numbers KHB 1676113, KHB 1676114, and KHB 1676115, respectively. The collected leaves were washed, dried at 40oC for 2 days, and ground into a fine powder using a mechanical grinder. The dried leaf materials collected from the three locations were pooled prior to extraction. The powdered material was stored in airtight containers at -20oC until extraction. To compare the effect of extraction solvent on macrophage-stimulating activity, powdered *R. fasciculatum* leaves were extracted with distilled water, 30% ethanol, 50% ethanol, or 70% ethanol. Briefly, dried leaf powder was mixed with each extraction solvent at a ratio of 1:20 (w/v) and extracted for 24 h at room temperature with gentle agitation. The resulting extracts were filtered through filter paper. The distilled water extract was freeze-dried. The ethanol extracts were concentrated by removing the solvent under reduced pressure at a temperature below 45°C and then were freeze-dried. The dried extracts were designated as RFL-DW, RFL-30E, RFL-50E, and RFL-70E, respectively. The extraction yields of RFL-DW20, RFL-30E, RFL-50E, and RFL-70E, calculated as the percentage of freeze-dried extract weight relative to the initial dry weight of the leaf powder, were 33.92%, 17.10%, 17.20%, and 16.98%, respectively.

To evaluate the effect of extraction temperature on macrophage-stimulating activity, dried leaf powder was extracted with distilled water at 20°C, 40°C, 60°C, or 80°C for 24 h. After extraction, each mixture was filtered, freeze-dried, and stored at -80°C until use. The extracts prepared at 20°C, 40°C, 60°C, and 80°C were designated as RFL-DW20, RFL-DW40, RFL-DW60, and RFL-DW80, respectively. To evaluate the effect of extraction time on macrophage-stimulating activity, dried leaf powder was extracted with distilled water at 20°C for 3, 6, 12, 16, 20, 24, or 48 h. Each extract was processed in the same manner as described above. Based on the NO-inducing activity in RAW264.7 cells, the water extract prepared at 20°C for 24 h, designated RFL-DW20, was selected for subsequent experiments because the activity reached a plateau by 24 h. For cell treatment, dried extracts were dissolved in sterile distilled water to prepare stock solutions. Working concentrations were freshly prepared by diluting the stock solutions with culture medium immediately before treatment. To evaluate the heat stability of RFL-DW20, the extract was subjected to autoclave treatment under two different conditions. For aqueous autoclave treatment, RFL-DW20 was dissolved in distilled water and autoclaved at 121°C for 15 min. For powder autoclave treatment, lyophilized RFL-DW20 powder was autoclaved under the same conditions and then dissolved in distilled water before use. Non-autoclaved RFL-DW20 was prepared in parallel and used as a control. LPS was also autoclaved under the same conditions and used as a heat-stable positive control. To prepare ethanol-precipitated and ethanol-soluble fractions, RFL-DW20 was dissolved in distilled water and mixed with cold ethanol to obtain a final ethanol concentration of 80%. The mixture was incubated at 4°C for 24 h to allow precipitation and then centrifuged at 12,000 × g for 30 min. The precipitate was collected, washed with ethanol, and freeze-dried to obtain the ethanol-precipitated fraction, designated RFL-DW20-EP. The supernatant was collected separately, concentrated to remove ethanol, and freeze-dried to obtain the ethanol-soluble supernatant fraction, designated RFL-DW20-ES. The recovery yield of each fraction was calculated as the dry weight of the recovered fraction relative to the dry weight of RFL-DW20 subjected to ethanol fractionation. From 1 g of RFL-DW20, 230 mg of RFL-DW20-EP and 700 mg of RFL-DW20-ES were recovered, corresponding to recovery yields of 23.0% and 70.0%, respectively, with an overall mass recovery of 93.0%. The crude RFL-DW20, RFL-DW20-EP, and RFL-DW20-ES were dissolved in sterile distilled water before use and compared for NO-inducing activity in RAW264.7 cells.

### Determination of Endotoxin Content

To directly assess possible endotoxin contamination in RFL-DW20, the endotoxin content was determined using the Quant-iT™ Endotoxin Detection Assay Kit (cat. no. Q32892, Invitrogen, Thermo Fisher Scientific, USA) according to the manufacturer’s instructions. RFL-DW20 was prepared at 100 μg/mL in endotoxin-free water. Endotoxin standards of 0, 0.01, 0.1, and 1.0 EU/mL were prepared using the endotoxin standard supplied with the kit, and endotoxin-free water was included as a negative control. All standards, controls, and samples were analyzed in triplicate. The mean fluorescence intensity of the 0 EU/mL standard was used for background correction. The lower detection limit under the assay conditions was 0.01 EU/mL. The complete endotoxin assay data, including the standard curve, negative control, replicate measurements, and mean ± SD values, are provided in Supplementary Fig. S1 and Supplementary Table S1.

### Cell Culture

Murine RAW264.7 macrophages were obtained from the American Type Culture Collection (cat. no. TIB-71, USA) and maintained in Dulbecco’s Modified Eagle Medium/Nutrient Mixture F-12 (DMEM/F-12, Cytiva, USA) supplemented with 10% fetal bovine serum (FBS, Cytiva, USA) and 1% penicillin-streptomycin. Cells were cultured at 37°C in a humidified incubator containing 5% CO_2_. For routine maintenance, RAW264.7 cells were subcultured before reaching full confluence to preserve stable macrophage-like characteristics. Because RAW264.7 cells are adherent macrophage-like cells, cells were detached gently using a cell scraper and reseeded in fresh complete medium. The culture medium was replaced every 2-3 days, and only cells showing normal morphology and stable growth were used for experiments.

### Cell viability Assay

Cell viability was evaluated using the MTT assay. RAW264.7 cells were seeded in 96-well plates at a density of 1 × 10^4^ cells/well and incubated overnight at 37°C in a humidified atmosphere containing 5% CO_2_. After cell attachment, the cells were treated with the indicated concentrations of each extract or RFL-DW20 for 24 h. For the comparison of extraction solvents, cells were treated with RFL-DW, RFL-30E, RFL-50E, or RFL-70E under both serum-containing and serum-free conditions. For the evaluation of RFL-DW20 cytotoxicity, cells were treated with RFL-DW20 at concentrations ranging from 12.5 to 100 μg/mL. After treatment, MTT solution was added to each well to a final concentration of 0.5 mg/mL, and the cells were further incubated for 4 h at 37°C. The culture medium was then carefully removed, and the formazan crystals formed in viable cells were dissolved in dimethyl sulfoxide (DMSO). The absorbance was measured at 570 nm using a microplate reader (SpectraMax M2, Molecular Devices, USA).

### Measurement of NO, PGE2, IL-1β, IL-6, and TNF-α Production

RAW264.7 cells were seeded in 24-well plates at a density of 1 × 10^5^ cells/well and allowed to attach overnight at 37°C in a humidified incubator containing 5% CO_2_. The cells were then treated with RFL-DW20 at the indicated concentrations for 24 h. In some experiments, cells were pretreated with PMB (20 μg/mL; LPS-neutralizing agent), TAK-242 (5 μM; TLR4 inhibitor), C29 (100 μM; TLR2 inhibitor), laminarin (250 μg/mL; Dectin-1-blocking ligand), mannan (1 mg/mL; mannose receptor-blocking ligand), PD98059 (20 μM), SB203580 (20 μM), SP600125 (20 μM), or BAY 11-7082 (20 μM) for 2 h before stimulation with RFL-DW20. LPS (1 μg/mL) was used as a positive control where indicated. After treatment, culture supernatants were collected and centrifuged at 1,000 × g for 5 min to remove cell debris. The clarified supernatants were used for the measurement of NO, PGE2, IL-6, and TNF-α production. NO production was determined by measuring nitrite accumulation in the culture medium using Griess reaction. Briefly, equal volumes of culture supernatant and Griess reagent were mixed in a 96-well plate and incubated at room temperature for 10 min in the dark. The absorbance was measured at 540 nm using a microplate reader (SpectraMax M2, Molecular Devices, USA). The levels of PGE2, IL-1β, IL-6, and TNF-α in the culture supernatants were measured using a Prostaglandin E2 Parameter Assay Kit (cat. no. KGE004B, R&D Systems, Minneapolis, MN, USA), Mouse IL-1β ELISA Kit (cat. no. BM6002-2, Invitrogen, USA), Mouse IL-6 ELISA Kit (cat. no. BMS6003-2, Invitrogen), and Mouse TNF alpha ELISA Kit (cat. no. BMS6007-3, Invitrogen) according to the manufacturers’ instructions, respectively.

### Neutral Red Uptake Assay

The phagocytic activity of RAW264.7 macrophages was evaluated using the neutral red uptake assay. RAW264.7 cells were seeded in 96-well plates at a density of 1 × 10^4^ cells/well and incubated overnight at 37°C in a humidified incubator containing 5% CO_2_. After attachment, the cells were treated with RFL-DW20 at the indicated concentrations for 24 h. In selected experiments, cells were pretreated with TAK-242 (5 μM; TLR4 inhibitor), SP600125 (20 μM), or BAY 11-7082 (20 μM) for 2 h before stimulation with RFL-DW20. After treatment, the culture medium was removed, and the cells were incubated with neutral red solution at a final concentration of 50 μg/mL for 2 h at 37°C. The cells were then washed gently with phosphate-buffered saline (PBS) to remove extracellular neutral red. The dye incorporated into the cells was extracted using a destaining solution composed of ethanol and acetic acid. After gentle shaking for 20 min at room temperature, the absorbance was measured at 540 nm using a microplate reader (SpectraMax M2, Molecular Devices, USA).

### Reverse Transcription-Polymerase Chain Reaction (RT-PCR)

The mRNA expression levels of iNOS, COX-2, IL-6, and TNF-α were determined by RT-PCR. RAW264.7 cells were seeded in 6-well plates at a density of 5 × 10^5^ cells/well and cultured overnight. After attachment, the cells were treated with RFL-DW20 at concentrations of 25, 50, and 100 μg/mL for 24 h. For inhibition experiments, cells were pretreated with TAK-242 (5 μM; TLR4 inhibitor) for 2 h and then stimulated with RFL-DW20. Total RNA was extracted using an RNeasy Mini Kit (cat. no. 74104, Qiagen, Germany) according to the manufacturer’s instructions. RNA concentration and purity were assessed by measuring the absorbance ratio at 260/280 nm using a GeneQuant 1300 spectrophotometer (Biochrom, USA). cDNA was synthesized from 1 μg of total RNA using a Verso cDNA Synthesis Kit (cat. no. AB1453B, Thermo Fisher Scientific) according to the manufacturer’s protocol. PCR was performed using a PCR Master Mix Kit (cat. no. M7502, Promega, USA) and gene-specific primers. The primer sequences used for iNOS, COX-2, IL-1β, IL-6, TNF-α or GAPDH in this study were as follows : iNOS: forward 5’-ttgtgcatcgacctaggctggaa-3’ and reverse 5’-gacctttcgcattagcatggaagc-3’, COX-2: forward 5’-gtactggctcatgctggacga-3’ and reverse 5’-caccatacactgccaggtcagcaa-3’, IL-1β: forward 5’-ggcaggcagtatcactcatt-3’ and reverse 5’-cccaaggccacaggtattt-3’, IL-6: forward 5′-gaggataccactcccaacagacc-3′ and reverse 5′-aagtgcatcatcgttgttcataca-3′, TNF-α: forward 5′-tggaactggcagaagaggca-3′ and reverse 5′-tgctcctccacttggtggtt-3′, GAPDH: forward 5’-ggactgtggtcatgagcccttcca-3’ and reverse 5’-actcacggcaaattcaacggcac-3’. The PCR conditions were as follows: 95°C for 5 min; 35 cycles of 95°C for 30 s, 58-60°C for 30 s, and 72°C for 30 s; followed by a final extension at 72°C for 5 min. PCR products were separated on a 1.5% agarose gel and visualized under UV light after staining with a nucleic acid staining reagent. The intensity of the visualized mRNA bands was quantified using UN-SCAN-IT gel software version 5.1 (Silk Scientific Inc., USA). The relative expression levels of target genes were calculated by normalizing the band intensity of each target gene to that of GAPDH.

### SDS-PAGE and Western Blot Analysis

RAW264.7 cells were seeded in 6-well plates at a density of 5 × 10^5^ cells/well and cultured overnight. For the analysis of iNOS and COX-2 expression, cells were treated with RFL-DW20 at concentrations of 25, 50, and 100 μg/mL for 24 h. For analysis of MAPK and NF-κB signaling, RAW264.7 cells were treated with RFL-DW20 (100 μg/mL) for the indicated time periods. The phosphorylation levels of ERK1/2, p38, JNK, and NF-κB p65 were examined at 0, 0.25, 0.5, 1, 3, and 6 h after treatment. To further evaluate canonical NF-κB activation, phosphorylation and degradation of IκBα were examined at 0, 5, 10, and 20 min after RFL-DW20 (100 μg/mL) treatment. For inhibition experiments, cells were pretreated with TAK-242 (5 μM; TLR4 inhibitor) for 2 h before stimulation with RFL-DW20 (100 μg/mL) for 30 min. After treatment, cells were washed with cold PBS and lysed with RIPA buffer (cat. no. 89901, Thermo Fisher Scientific) supplemented with protease and phosphatase inhibitors. The lysates were kept on ice for 30 min and centrifuged at 12,000 × g for 30 min at 4°C. Protein concentrations were measured using a Pierce™ BCA Protein Assay Kit (cat. no. 23225, Thermo Fisher Scientific). Equal amounts of protein (30 μg/well) were separated on 10% SDS-polyacrylamide gels and transferred to a nitrocellulose membrane (cat. no. 88018, Thermo Fisher Scientific). The membranes were blocked with 5% skim milk in TBST for 1 h at room temperature. The membranes were then incubated overnight at 4°C with primary antibodies specific for iNOS, COX-2, phospho-JNK, JNK, phospho-NF-κB p65, NF-κB p65, and β-actin. After washing three times with TBST, the membranes were incubated with HRP-conjugated secondary antibodies for 1 h at room temperature. Immunoreactive bands were visualized using an ECL Select Western Blotting Detection Reagent (cat. no. RPN2232, Cytiva, USA) and detected with an LI-COR C-DiGit Blot Scanner (LI-COR Biosciences, USA). The intensity of the visualized protein bands was quantified using UN-SCAN-IT gel software version 5.1 (Silk Scientific Inc.). iNOS and COX-2 levels were normalized to β-actin. Phosphorylated ERK1/2, p38, JNK, and NF-κB p65 levels were normalized to their corresponding total proteins. Phosphorylated IκBα and total IκBα levels were normalized to β-actin.

### Determination of Total Polyphenol, Flavonoid, and Carbohydrate Contents

The chemical compositions of RFL-DW20, RFL-DW20-EP, and RFL-DW20-ES were characterized by measuring total polyphenol, flavonoid, and carbohydrate contents. RFL-DW20 was dissolved in distilled water at an appropriate concentration and used for each colorimetric assay. The total polyphenol content was determined using the Folin-Ciocalteu method. Briefly, RFL-DW20 solution was mixed with Folin-Ciocalteu reagent and incubated at room temperature. After the addition of sodium carbonate solution, the reaction mixture was further incubated in the dark. The absorbance was measured at 765 nm using a microplate reader (SpectraMax M2, Molecular Devices, USA). Gallic acid was used as the standard. The total flavonoid content was measured using the aluminum chloride colorimetric method. RFL-DW20 solution was mixed with aluminum chloride reagent and incubated at room temperature. The absorbance was measured at 415 nm using a microplate reader (SpectraMax M2, Molecular Devices). Quercetin was used as the standard. The total carbohydrate content was determined using the phenol-sulfuric acid method. RFL-DW20 solution was mixed with phenol solution, followed by the addition of concentrated sulfuric acid. After incubation at room temperature, the absorbance was measured at 490 nm using a microplate reader. Mannose was used as the standard.

### HPLC Chromatographic Fingerprint Analysis

The chromatographic profiles of RFL-DW20, RFL-DW20-EP, and RFL-DW20-ES were analyzed using a Waters Alliance 2695 HPLC system equipped with a photodiode array (PDA) detector. Separation was performed on a SunFire C18 column (5 μm, 100 Å, 250 × 4.6 mm) maintained at 25°C. The mobile phases consisted of distilled water (A) and acetonitrile (C). The gradient elution program was as follows: 0-5 min, 95% A/5% B; 20 min, 85% A/15% B; 35 min, 75% A/25% B; 45 min, 55% A/45% B; 50-55 min, 10% A/90% B; and 60 min, 95% A/5% B. The flow rate was 1.0 mL/min, and the injection volume was 10 μL. Chromatographic profiles were monitored at 254 nm using the PDA detector. RFL-DW20, RFL-DW20-EP, and RFL-DW20-ES were dissolved in distilled water at 10 mg/mL and analyzed under identical chromatographic conditions.

### Statistical Analysis

All experiments were repeated at least three times. Statistical analysis was performed using GraphPad Prism version 5.0 (GraphPad Software, Inc.) and data are presented as the mean ± standard deviation. Data were analyzed using one-way analysis of variance followed by Bonferroni's post hoc test. *P* < 0.05 was considered to indicate a statistically significant difference

## Results

### Selection of Extraction Conditions for the Immunostimulatory Activity of *R. fasciculatum* Leaves in RAW264.7 Macrophages

**Fig. 1:**
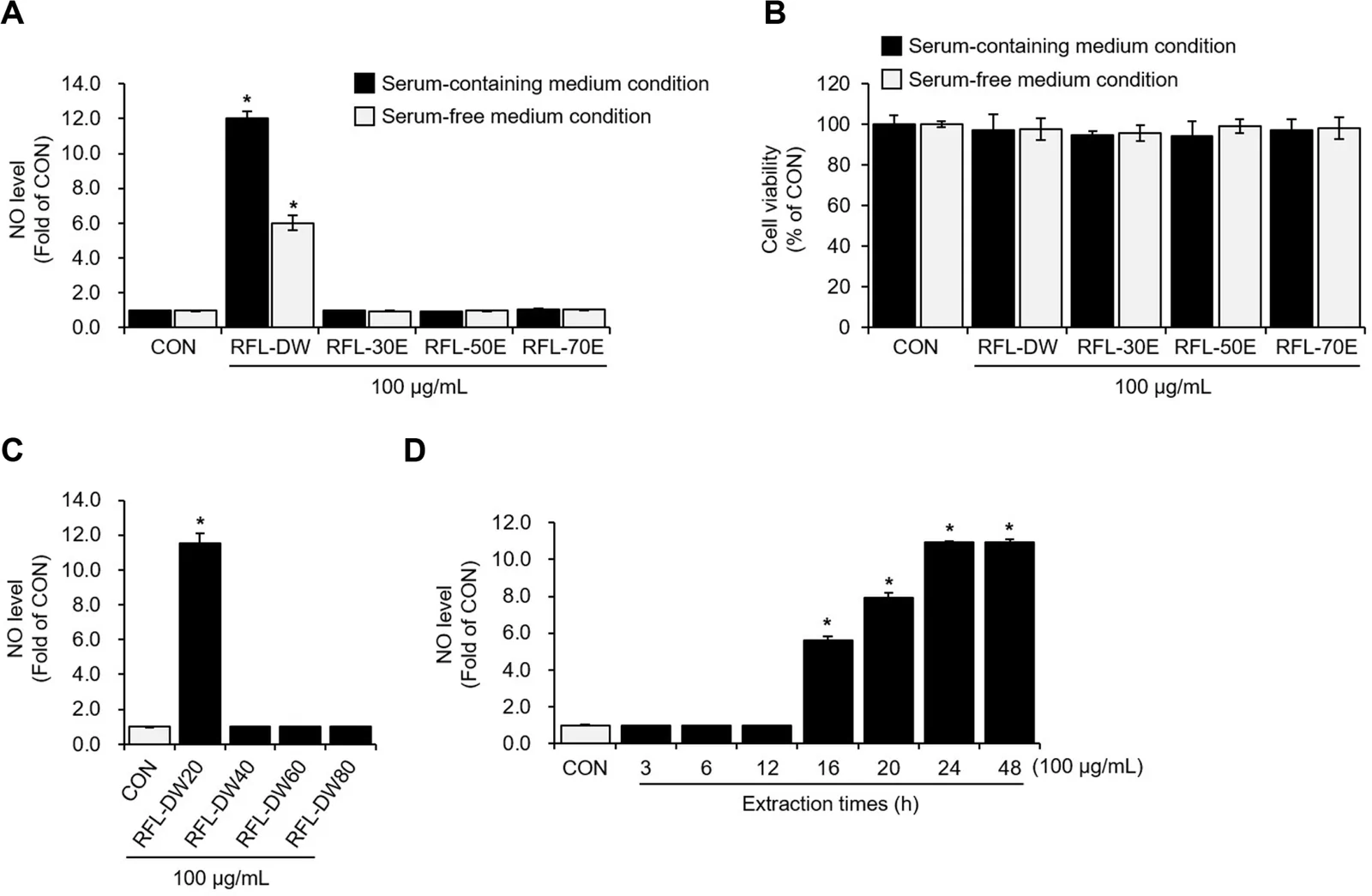
Effects of extraction conditions on the macrophage-activating activity of *R. fasciculatum* leaves. (**A**) RAW264.7 cells were treated with *R. fasciculatum* leaf water extract (RFL-DW), 30% ethanol extract (RFL-30E), 50% ethanol extract (RFL-50E), or 70% ethanol extract (RFL-70E) under serum-containing or serum-free conditions for 24 h, and NO production was measured using the Griess assay (**B**) Cell viability was evaluated using MTT assay after treatment with RFL-DW, RFL-30E, RFL-50E, or RFL-70E under serum-containing or serum-free conditions for 24 h. (**C**) RAW264.7 cells were treated with water extracts prepared at 20°C, 40°C, 60°C, or 80°C for 24 h, designated RFL-DW20, RFL-DW40, RFL-DW60, and RFL-DW80, respectively, and NO production was determined using the Griess assay. (**D**) RAW264.7 cells were treated with water extracts prepared at 20°C for 3, 6, 12, 16, 20, 24, or 48 h, and NO production was measured using the Griess assay. *P < 0.05 vs. CON (untreated group).

To identify the extract fraction responsible for the macrophage-activating activity of *R. fasciculatum* leaves, RAW264.7 cells were treated with water extract (RFL-DW), 30% ethanol extract (RFL-30E), 50% ethanol extract (RFL-50E), or 70% ethanol extract (RFL-70E), and NO production was measured in both serum-containing and serum-free media. Among the tested extracts, only RFL-DW markedly increased NO production in RAW264.7 cells under both culture conditions, whereas RFL-30E, RFL-50E, and RFL-70E failed to induce NO production (Fig. 1A). In addition, RFL-DW20 showed an extraction yield of 33.92%, which was higher than those of RFL-30E (17.10%), RFL-50E (17.20%), and RFL-70E (16.98%). Thus, among the solvent extracts tested, the water extract exhibited both the highest extraction yield and the strongest NO-inducing activity. This result indicates that the macrophage-activating component(s) of *R. fasciculatum* leaves are more efficiently recovered in the water extract than in ethanol-containing extracts. To exclude the possibility that the changes in NO production were associated with cytotoxicity, cell viability was examined under the same experimental conditions. None of the extracts, including RFL-DW, RFL-30E, RFL-50E, and RFL-70E, affected the viability of RAW264.7 cells in either serum-containing or serum-free media (Fig. 1B). These data suggest that RFL-DW-induced NO production was not attributable to extract-mediated cytotoxic stress but rather reflected macrophage activation. Because RFL-DW showed the strongest NO-inducing activity, the effect of water extraction temperature on macrophage-stimulating activity was further evaluated. RAW264.7 cells were treated with water extracts prepared at 20°C, 40°C, 60°C, or 80°C, designated RFL-DW20, RFL-DW40, RFL-DW60, and RFL-DW80, respectively. Among these extracts, only RFL-DW20 induced NO production, whereas RFL-DW40, RFL-DW60, and RFL-DW80 showed no NO-inducing activity (Fig. 1C). These findings suggest that the active constituent(s) responsible for macrophage activation may be preferentially extracted at low temperatures or may be sensitive to elevated extraction temperatures. Next, to further characterize the effect of extraction time on macrophage-stimulating activity, *R. fasciculatum* leaves were extracted with water at 20°C for 3, 6, 12, 16, 20, 24, or 48 h, and the NO-inducing activity of each extract was compared. Extracts prepared for up to 12 h showed little or no change in NO production. In contrast, NO-inducing activity became evident after 16 h of extraction and increased progressively, reaching approximately 5.6-, 7.9-, and 10.9-fold over the untreated control at 16, 20, and 24 h, respectively (Fig. 1D). Extension of the extraction time to 48 h did not further increase the activity, which remained comparable to that observed at 24 h. These results demonstrate a delayed time-dependent increase in macrophage-stimulating activity after 12 h of extraction, with the activity reaching a plateau by 24 h. Therefore, the extract prepared at 20°C for 24 h (RFL-DW20) was selected for subsequent experiments.

### RFL-DW20 Enhances NO and PGE2 Production and Upregulates iNOS and COX-2 Expression in RAW264.7 Macrophages

**Fig. 2:**
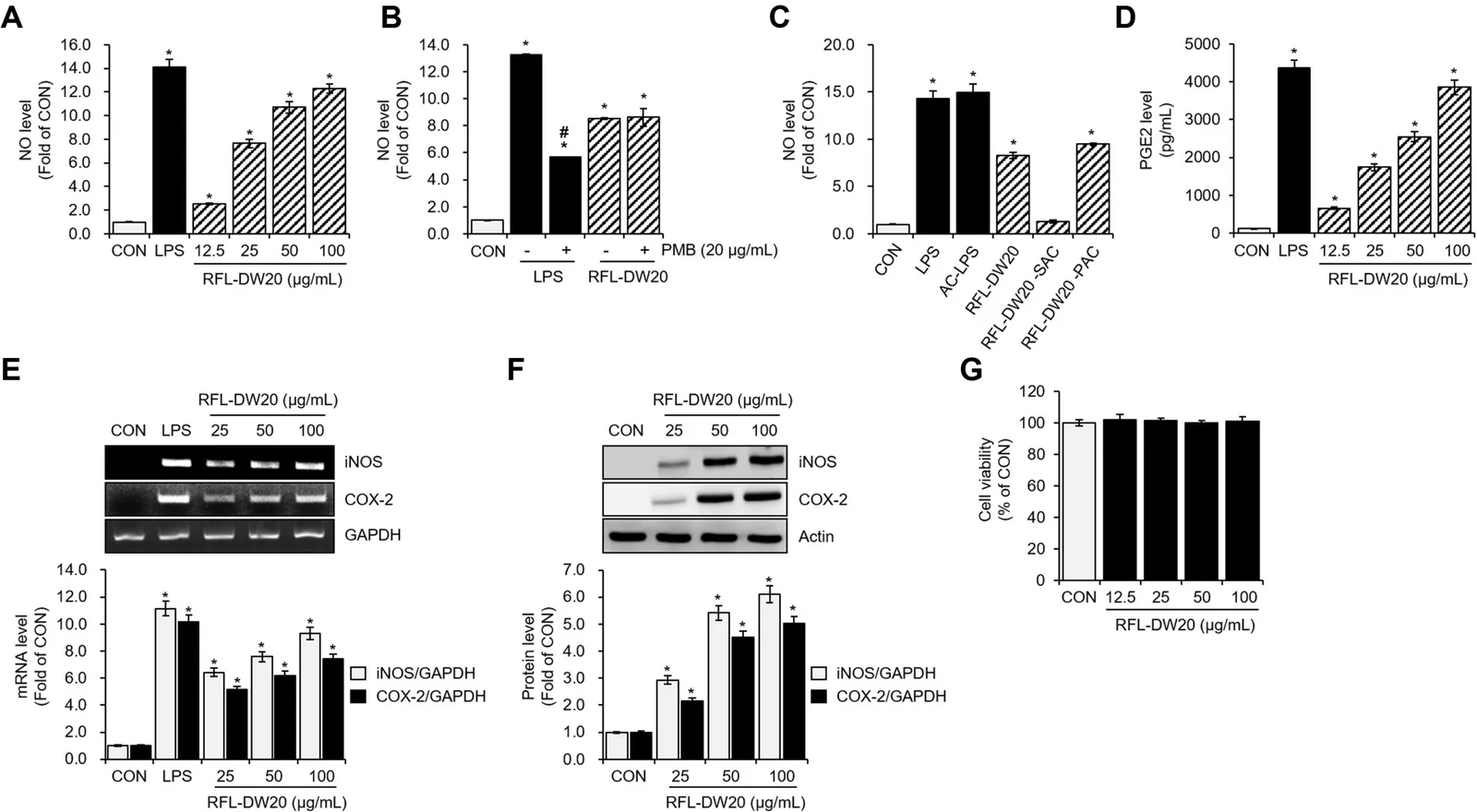
RFL-DW20 increases NO and PGE2 production and upregulates iNOS and COX-2 expressions in RAW264.7 macrophages. (**A**) RAW264.7 cells were treated with RFL-DW20 at concentrations of 12.5-100 μg/mL, and NO production was measured using the Griess assay. (**B**) Cells were treated with LPS (1 μg/mL) or RFL-DW20 (100 μg/mL) in the presence or absence of PMB (20 μg/mL), and NO production was analyzed using the Griess assay. (**C**) LPS (1 μg/mL) and RFL-DW20 (100 μg/mL) were subjected to autoclave treatment, and their NO-inducing activities were compared using the Griess assay. For RFL-DW20, autoclave treatment was performed in either aqueous solution or powder form. (**D**) PGE2 production was measured in RAW264.7 cells treated with RFL-DW20 at concentrations of 12.5-100 μg/mL for 24 h. (**E**) The mRNA expression levels of iNOS and COX-2 were analyzed by RT-PCR after treatment with RFL-DW20 at concentrations of 25-100 μg/mL for 24 h. (**F**) The protein expression levels of iNOS and COX-2 were examined by Western blot analysis after treatment with RFL-DW20 at concentrations of 25-100 μg/mL for 24 h. (**G**) Cell viability was evaluated after treatment with RFL-DW20 at concentrations of 12.5-100 μg/mL. *P < 0.05 vs. CON (untreated group).

Based on the extraction-condition comparisons shown in Fig. 1, RFL-DW20 was selected for subsequent experiments. To evaluate the macrophage-activating effect of RFL-DW20, RAW264.7 cells were treated with various concentrations of RFL-DW20, and NO production was measured. RFL-DW20 increased NO production in a concentration-dependent manner at concentrations ranging from 12.5 to 100 μg/mL (Fig. 2A), indicating that RFL-DW20 effectively stimulates NO production in RAW264.7 macrophages.

Because endotoxin contamination can induce NO production in macrophages, PMB, an endotoxin-neutralizing agent, was used to determine whether the NO-inducing effect of RFL-DW20 was attributable to endotoxin contamination. As expected, PMB markedly reduced LPS-induced NO production. In contrast, PMB did not suppress RFL-DW20-induced NO production (Fig. 2B). These results suggest that the NO-inducing activity of RFL-DW20 was not due to endotoxin contamination. To further directly assess possible endotoxin contamination, the endotoxin content of RFL-DW20 was quantified using a fluorescent endotoxin detection assay. The log-transformed endotoxin standard curve showed a strong correlation (r = 0.9975). The mean fluorescence intensity of RFL-DW20 at 100 μg/mL was 8.11 ± 0.13 RFU, which was comparable to that of the 0 EU/mL standard (8.01 ± 0.12 RFU) and endotoxin-free water (8.41 ± 0.29 RFU), but markedly lower than that of the lowest positive standard (0.01 EU/mL; 15.88 ± 0.43 RFU). Accordingly, the endotoxin level in RFL-DW20 was determined to be below 0.01 EU/mL (Supplementary Fig. S1 and Table S1). These results further indicate that the NO-inducing activity of RFL-DW20 is unlikely to be attributable to endotoxin contamination. To further distinguish the activity of RFL-DW20 from that of LPS, the effects of autoclaving were examined. Autoclaved LPS retained its NO-inducing activity, showing a response comparable to that of non-autoclaved LPS. In contrast, RFL-DW20 lost its NO-inducing activity when autoclaved in aqueous solution. However, when RFL-DW20 was autoclaved in powder form, its NO-inducing activity was maintained at a level similar to that of non-autoclaved RFL-DW20 (Fig. 2C). These findings indicate that the macrophage-activating activity of RFL-DW20 differs from that of LPS and may be affected by heat treatment under aqueous conditions. Next, we examined whether RFL-DW20 also regulates PGE2 production, another macrophage-derived immune mediator. RFL-DW20 increased PGE2 production in a concentration-dependent manner at concentrations ranging from 12.5 to 100 μg/mL (Fig. 2D). Consistent with the increases in NO and PGE2 production, RFL-DW20 also upregulated the mRNA expression levels of iNOS and COX-2 in a concentration-dependent manner at concentrations of 25-100 μg/mL (Fig. 2E). In addition, Western blot analysis showed that RFL-DW20 increased the protein expression levels of iNOS and COX-2 in a concentration-dependent manner (Fig. 2F). These results demonstrate that RFL-DW20 stimulates NO and PGE2 production through transcriptional and translational upregulation of iNOS and COX-2 in RAW264.7 macrophages. To determine whether the immunostimulatory effects of RFL-DW20 were associated with cytotoxicity, cell viability was assessed after treatment with RFL-DW20. RFL-DW20 did not affect RAW264.7 cell viability at concentrations ranging from 12.5 to 100 μg/mL (Fig. 2G). Therefore, the RFL-DW20-induced increases in NO, PGE2, iNOS, and COX-2 were not caused by cytotoxic effects but reflected macrophage activation.

### RFL-DW20 Modulates Cytokine Responses and Enhances Neutral Red Uptake in RAW264.7 Macrophages

**Fig. 3:**
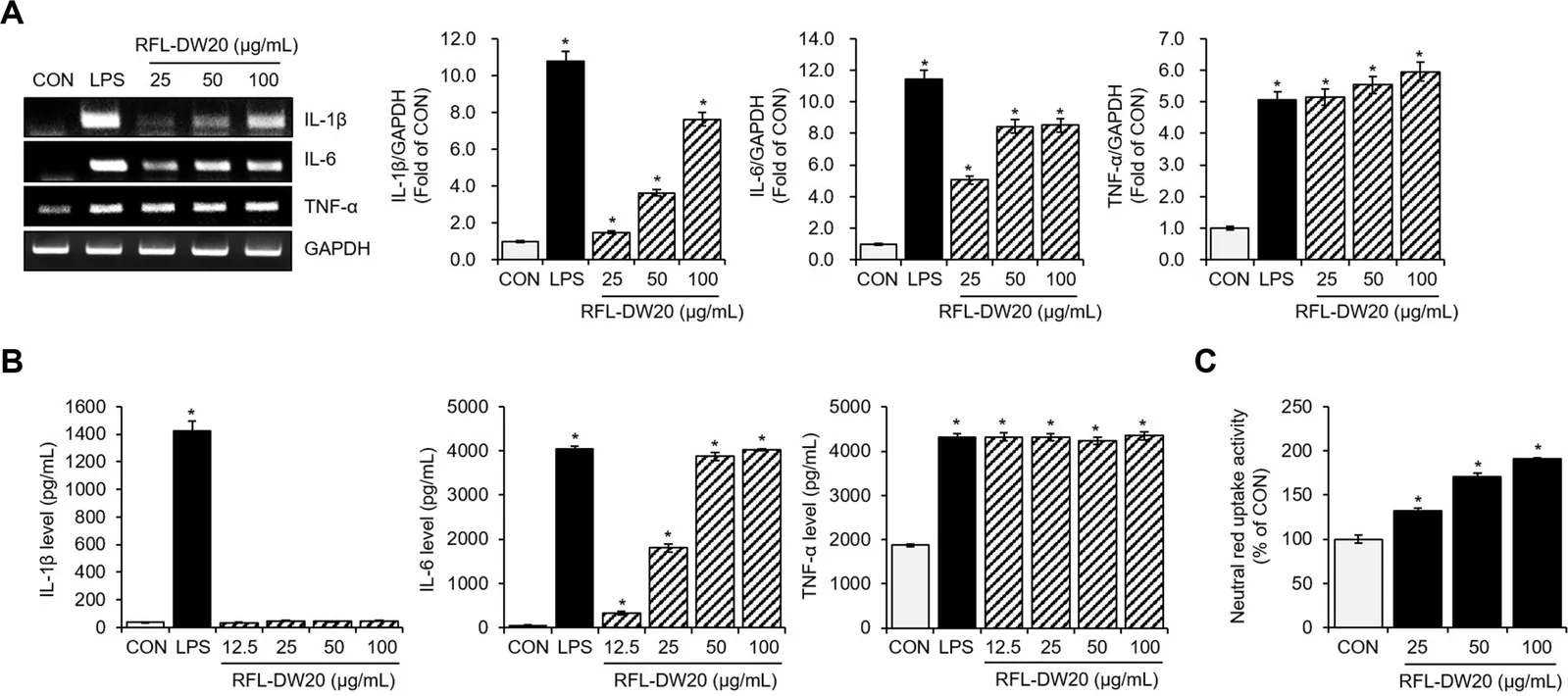
RFL-DW20 modulates cytokine responses and enhances neutral red uptake in RAW264.7 macrophages. (**A**) RAW264.7 cells were treated with RFL-DW20 at concentrations of 25-100 μg/mL for 24 h, and the mRNA expression levels of IL-1β, IL-6, and TNF-α were analyzed by RT-PCR. (**B**) RAW264.7 cells were treated with RFL-DW20 at concentrations of 12.5-100 μg/mL for 24 h, and the extracellular levels of IL-1β, IL-6, and TNF-α in the culture medium were measured by ELISA Kit. (**C**) Neutral red uptake was evaluated in RAW264.7 cells treated with RFL-DW20 at concentrations of 25-100 μg/mL for 24 h. *P < 0.05 vs. CON (untreated group).

To further characterize the cytokine response induced by RFL-DW20, we examined the expression and secretion of IL-1β, IL-6, and TNF-α in RAW264.7 macrophages. RFL-DW20 increased the mRNA expression levels of IL-1β, IL-6, and TNF-α (Fig. 3A). We next examined the extracellular levels of these cytokines in the culture medium. RFL-DW20 increased IL-6 secretion in a concentration-dependent manner at concentrations ranging from 12.5 to 100 μg/mL, whereas TNF-α secretion was elevated but remained relatively similar across the tested concentrations (Fig. 3B). In contrast, despite the marked increase in IL-1β mRNA expression, extracellular IL-1β levels were not significantly altered by RFL-DW20 treatment. LPS, used as a positive control, markedly increased extracellular IL-1β levels. These findings indicate that RFL-DW20 induces cytokine-specific transcriptional and secretory responses, with transcriptional induction of IL-1β not accompanied by a corresponding increase in its extracellular level under the present experimental conditions. Because macrophage activation is closely associated with increased phagocytic activity [6], we next evaluated the effect of RFL-DW20 on neutral red uptake. RFL-DW20 significantly enhanced neutral red uptake in a concentration-dependent manner at concentrations ranging from 25 to 100 μg/mL (Fig. 3C). These results demonstrate that RFL-DW20 induces distinct cytokine responses and enhances the functional activation of RAW264.7 macrophages.

### TLR4 Is Required for RFL-DW20-Mediated Macrophage Activation in RAW264.7 Cells

**Fig. 4:**
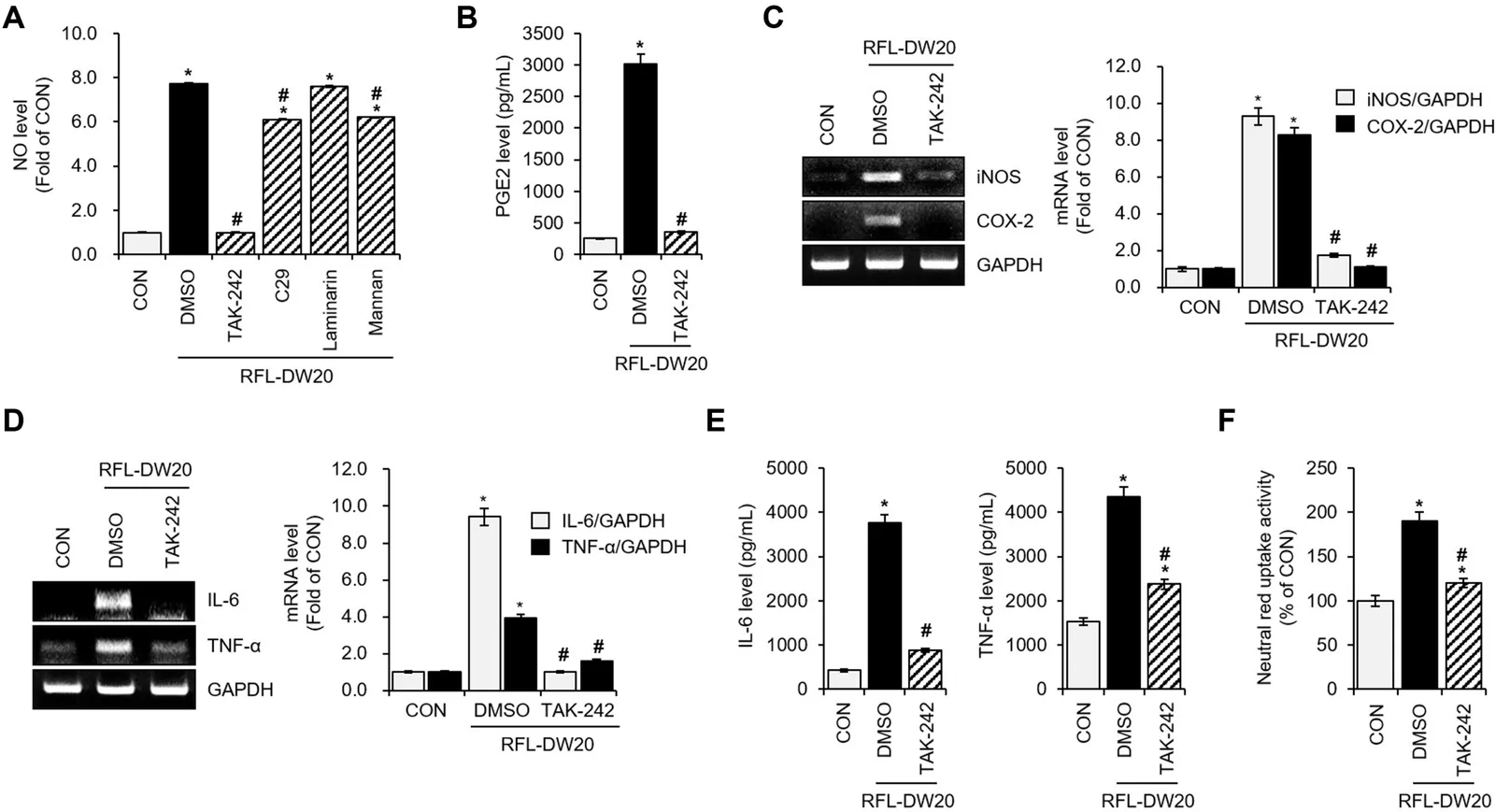
TLR4 is involved in RFL-DW20-mediated macrophage activation in RAW264.7 cells. (**A**) RAW264.7 cells were pretreated with TAK-242 (5 μM), C29 (100 μM), laminarin (250 μg/mL), or mannan (1 mg/mL) for 2 h, followed by stimulation with RFL-DW20 (100 μg/mL) for 24 h, and NO production was measured using the Griess assay. (**B**) Cells were pretreated with TAK-242 (5 μM) for 2 h and then stimulated with RFL-DW20 (100 μg/mL) for 24 h, and PGE2 production was analyzed using ELISA Kit. (**C**) The mRNA expression levels of iNOS and COX-2 were analyzed in cells pretreated with TAK-242 (5 μM) for 2 h before RFL-DW20 (100 μg/mL) stimulation for 24 h. (**D**) The mRNA expression levels of IL-6 and TNF-α were determined after TAK-242 (5 μM) pretreatment for 2 h and RFL-DW20 (100 μg/mL) stimulation for 24 h. (**E**) The secretion levels of IL-6 and TNF-α were measured using ELISA Kit in culture supernatants from cells treated with TAK-242 (5 μM) and RFL-DW20 (100 μg/mL). (**F**) Neutral red uptake was evaluated in cells pretreated with TAK-242 (5 μM) for 2 h before RFL-DW20 (100 μg/mL) stimulation for 24 h. *P < 0.05 vs. CON (untreated group). ^#^*P*<0.05 vs. DMSO (group treated with RFL-DW20 alone).

To identify the receptor involved in RFL-DW20-induced macrophage activation, RAW264.7 cells were pretreated with inhibitors targeting major pattern-recognition receptors, including TAK-242, C29, laminarin, and mannan, followed by stimulation with RFL-DW20. Among these treatments, TAK-242, a TLR4 signaling inhibitor, markedly suppressed RFL-DW20-induced NO production (Fig. 4A). C29 and mannan slightly reduced RFL-DW20-mediated NO production, whereas laminarin did not inhibit the NO-inducing effect of RFL-DW20. These results suggest that TLR4 is the major receptor involved in RFL-DW20-induced NO production in RAW264.7 macrophages. Based on the strong inhibitory effect of TAK-242 on RFL-DW20-induced NO production, we next examined whether TLR4 signaling is required for the production of other macrophage-derived immune mediators. TAK-242 markedly inhibited RFL-DW20-induced PGE2 production (Fig. 4B). In addition, TAK-242 strongly suppressed the RFL-DW20-mediated upregulation of iNOS and COX-2 mRNA expression (Fig. 4C), indicating that TLR4 signaling contributes to the transcriptional regulation of enzymes responsible for NO and PGE2 production in RFL-DW20-treated RAW264.7 cells. We further investigated whether TLR4 is involved in RFL-DW20-induced cytokine expression and secretion. TAK-242 markedly attenuated the RFL-DW20-induced mRNA expression of IL-6 and TNF-α (Fig. 4D). Consistent with these mRNA expression data, TAK-242 also strongly reduced the secretion of IL-6 and TNF-α in RFL-DW20-treated RAW264.7 cells (Fig. 4E). These findings demonstrate that RFL-DW20-induced IL-6 and TNF-α responses are largely dependent on TLR4 signaling. Finally, to determine whether TLR4 signaling also contributes to the functional activation of macrophages by RFL-DW20, neutral red uptake was evaluated in the presence or absence of TAK-242. TAK-242 markedly inhibited the increase in neutral red uptake induced by RFL-DW20 (Fig. 4F). Taken together, these results indicate that TLR4 plays a critical role in RFL-DW20-mediated macrophage activation, including NO and PGE2 production, iNOS and COX-2 expression, IL-6 and TNF-α production, and enhancement of neutral red uptake in RAW264.7 cells.

### JNK and NF-κB Signaling Are Involved in RFL-DW20-Mediated Macrophage Activation

**Fig. 5:**
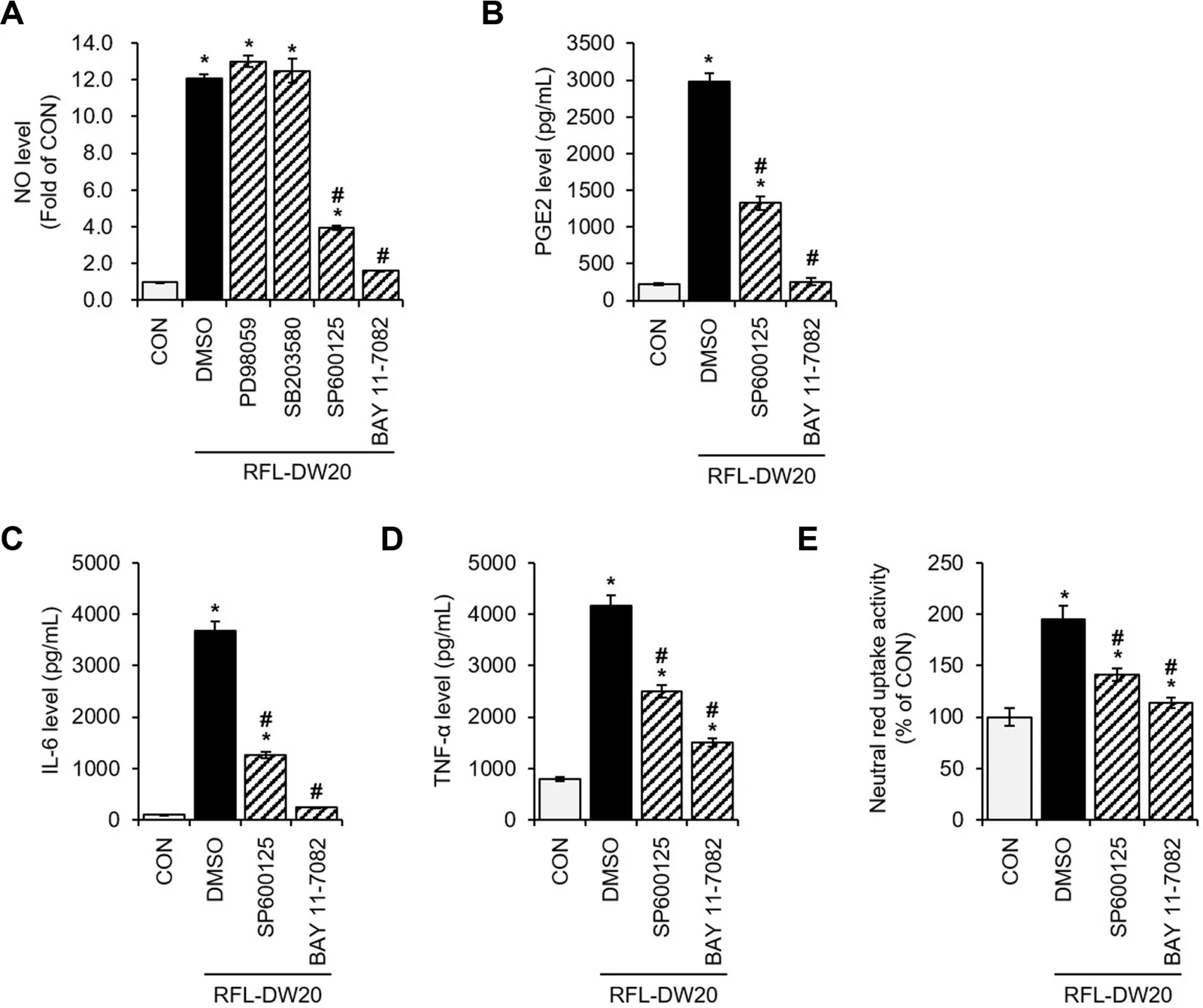
JNK and NF-κB signaling contribute to RFL-DW20-induced macrophage activation. (**A**) RAW264.7 cells were pretreated with PD98059 (20 μM), SB203580 (20 μM), SP600125 (20 μM), or BAY 11-7082 (20 μM) for 2 h, followed by stimulation with RFL-DW20 (100 μg/mL) for 24 h, and NO production was measured using the Griess assay. (**B**) PGE2 production was analyzed using ELISA Kit in cells pretreated with SP600125 (20 μM) or BAY 11-7082 (20 μM) for 2 h before RFL-DW20 (100 μg/mL) stimulation for 24 h. (**C** and **D**) The secretion levels of IL-6 and TNF-α were measured using ELISA Kit in cells pretreated with SP600125 (20 μM) or BAY 11-7082 (20 μM) for 2 h and then stimulated with RFL-DW20 (100 μg/mL) for 24 h. (**E**) Neutral red uptake was evaluated after pretreatment with SP600125 (20 μM) or BAY 11-7082 (20 μM) for 2 h and subsequent stimulation with RFL-DW20 (100 μg/mL) for 24 h. *P < 0.05 vs. CON (untreated group). #*P* < 0.05 vs. DMSO (group treated with RFL-DW20 alone).

To determine which downstream signaling pathways are required for RFL-DW20-induced macrophage activation, RAW264.7 cells were pretreated with pharmacological inhibitors targeting ERK1/2, p38, JNK, and NF-κB, including PD98059, SB203580, SP600125, and BAY 11-7082, respectively, followed by stimulation with RFL-DW20. PD98059 and SB203580 did not suppress RFL-DW20-induced NO production, whereas SP600125 and BAY 11-7082 markedly inhibited the NO-inducing effect of RFL-DW20 (Fig. 5A). These results suggest that JNK and NF-κB signaling are functionally required for RFL-DW20-induced NO production, whereas ERK1/2 and p38 signaling are dispensable for this response under the present experimental conditions. Based on the inhibitory effects of SP600125 and BAY 11-7082 on NO production, we further investigated whether JNK and NF-κB signaling also regulate the production of other immune mediators induced by RFL-DW20. Both SP600125 and BAY 11-7082 markedly reduced RFL-DW20-induced PGE2 production (Fig. 5B). In addition, SP600125 and BAY 11-7082 significantly attenuated the secretion of IL-6 and TNF-α in RFL-DW20-treated RAW264.7 cells (Fig. 5C and 5D). These findings indicate that JNK and NF-κB signaling contribute to the production of multiple macrophage-derived immune mediators in response to RFL-DW20. Finally, we examined whether JNK and NF-κB signaling are also involved in the functional activation of macrophages by RFL-DW20. RFL-DW20-enhanced neutral red uptake was markedly suppressed by pretreatment with either SP600125 or BAY 11-7082 (Fig. 5E). Taken together, these results demonstrate that activation of JNK and NF-κB signaling is required for RFL-DW20-mediated macrophage activation, including NO, PGE2, IL-6, and TNF-α production, as well as enhanced neutral red uptake.

### RFL-DW20 Activates MAPK and Canonical NF-κB Signaling in RAW264.7 Macrophages

**Fig. 6:**
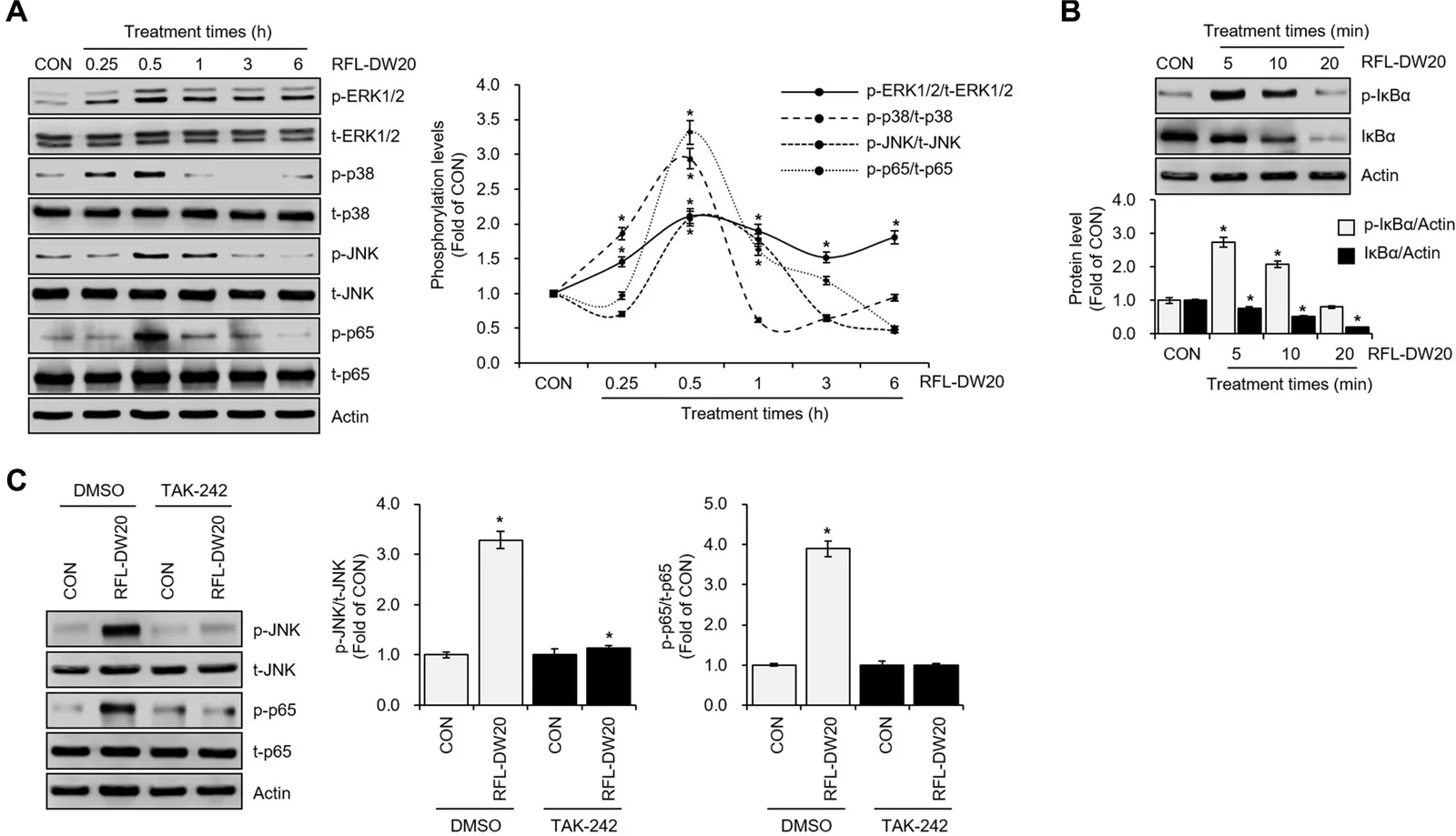
RFL-DW20 activates MAPK and canonical NF-κB signaling in RAW264.7 macrophages. (**A**) RAW264.7 cells were treated with RFL-DW20 (100 μg/mL) for the indicated time periods, and the phosphorylation levels of ERK1/2, p38, JNK, and NF-κB p65 were examined by Western blot analysis. Phosphorylated proteins were normalized to their respective total proteins. (**B**) RAW264.7 cells were treated with RFL-DW20 (100 μg/mL) for 5, 10, or 20 min, and phosphorylation and degradation of IκBα were examined by Western blot analysis. p-IκBα and total IκBα were normalized to β-actin. (**C**) RAW264.7 cells were pretreated with TAK-242 (5 μM) for 2 h before stimulation with RFL-DW20 (100 μg/mL) for 30 min. The phosphorylation levels of JNK and NF-κB p65 were analyzed by Western blotting and normalized to their respective total proteins. *P < 0.05 vs. CON (untreated group).

To further characterize the signaling pathways activated by RFL-DW20, RAW264.7 cells were treated with RFL-DW20, and the phosphorylation levels of ERK1/2, p38, JNK, and NF-κB p65 were examined over time. RFL-DW20 increased the phosphorylation of ERK1/2, p38, JNK, and p65, although their temporal activation profiles differed (Fig. 6A). Phosphorylation of p38, JNK, and p65 was markedly increased within 15-30 min, with prominent activation observed at 30 min, whereas ERK1/2 phosphorylation showed a relatively sustained increase following RFL-DW20 treatment. These findings indicate that RFL-DW20 activates multiple MAPK pathways as well as NF-κB signaling. Because inhibitor experiments showed that PD98059 and SB203580 did not suppress RFL-DW20-induced NO production (Fig. 5A), the observed phosphorylation of ERK1/2 and p38 indicates that these pathways are activated by RFL-DW20 but are not required for its NO-inducing effect under the present experimental conditions. To further examine activation of the canonical NF-κB pathway, phosphorylation and degradation of IκBα were analyzed. RFL-DW20 rapidly increased IκBα phosphorylation at 5 min, which was accompanied by a time-dependent reduction in total IκBα levels (Fig. 6B). These findings provide additional evidence supporting activation of canonical NF-κB signaling by RFL-DW20. Finally, because TLR4 was identified as a major receptor involved in RFL-DW20-mediated macrophage activation, we examined whether RFL-DW20-induced JNK and p65 phosphorylation was dependent on TLR4 signaling. Pretreatment with TAK-242 (TLR4 inhibitor) markedly suppressed RFL-DW20-induced phosphorylation of both JNK and p65 (Fig. 6C). Taken together with the pharmacological inhibitor data in Fig. 5, these results suggest that RFL-DW20 activates multiple MAPK pathways, while JNK and canonical NF-κB signaling play major functional roles in its macrophage-stimulating effects downstream of TLR4.

### Chemical Composition and Enrichment of Macrophage-Stimulating Activity in the Ethanol-Precipitated Fraction of RFL-DW20

**Fig. 7:**
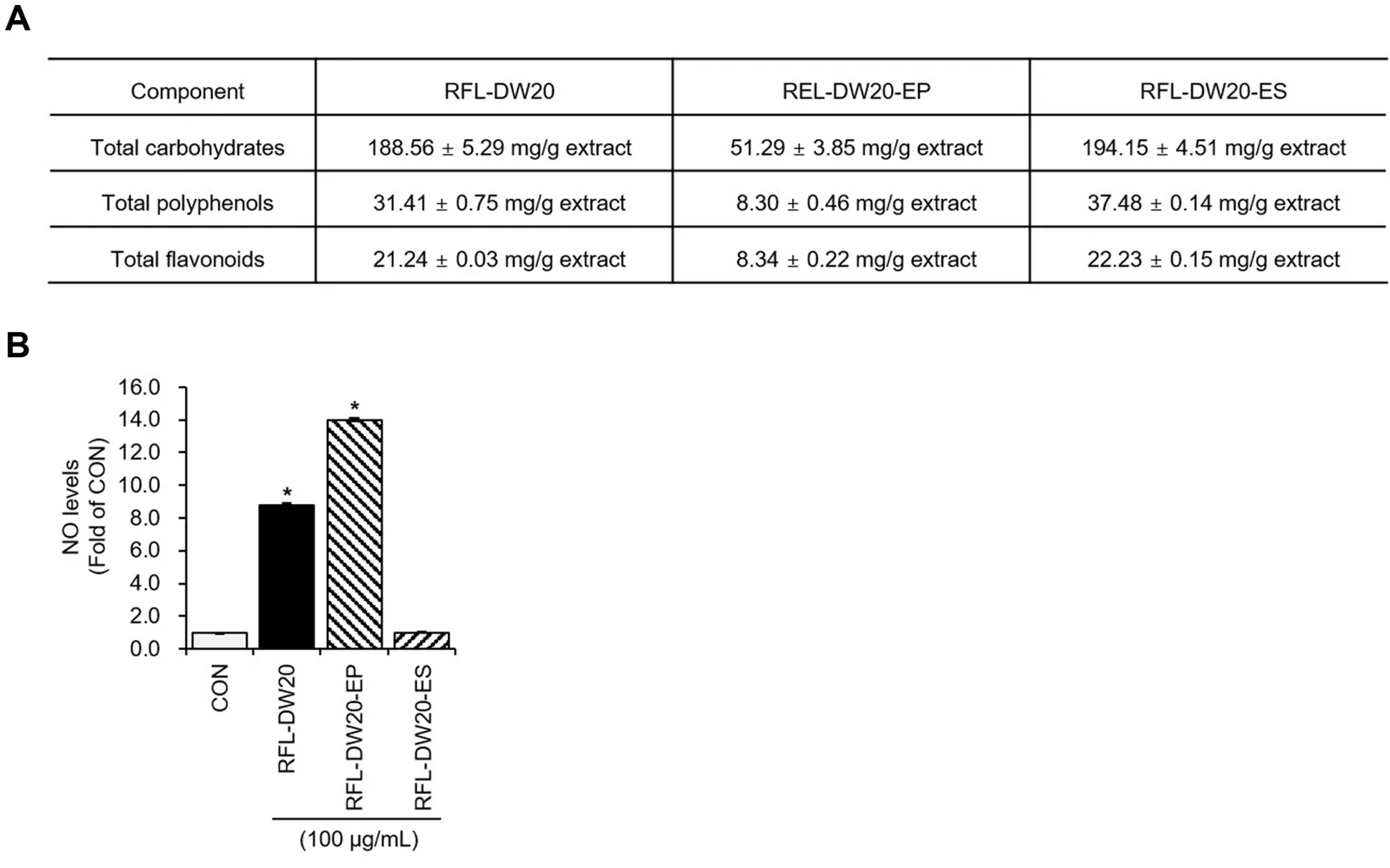
Chemical composition and enrichment of macrophage-stimulating activity in the ethanol-precipitated fraction of RFL-DW20. (**A**) Total polyphenol, flavonoid, and carbohydrate of RFL-DW20, RFL-DW20-EP, and RFL-DW20-ES were determined using colorimetric assays. (**B**) RFL-DW20 was separated into an ethanol-precipitated fraction (RFL-DW20-EP) and an ethanol-soluble supernatant fraction (RFL-DW20-ES), and their NO-inducing activities were compared with that of crude RFL-DW20 in RAW264.7 cells. *P < 0.05 vs. CON (untreated group).

To further characterize the chemical constituents associated with the macrophage-stimulating activity of RFL-DW20, the chemical compositions of crude RFL-DW20, RFL-DW20-EP, and RFL-DW20-ES were compared. The total carbohydrate contents of RFL-DW20, RFL-DW20-EP, and RFL-DW20-ES were 188.56 ± 5.29, 51.29 ± 3.85, and 194.15 ± 4.51 mg/g extract, respectively. The corresponding total polyphenol contents were 31.41 ± 0.75, 8.30 ± 0.46, and 37.48 ± 0.14 mg/g extract, while the total flavonoid contents were 21.24 ± 0.03, 8.34 ± 0.22, and 22.23 ± 0.15 mg/g extract, respectively (Fig. 7A). Ethanol fractionation of RFL-DW20 yielded 23.0% RFL-DW20-EP and 70.0% RFL-DW20-ES, with an overall mass recovery of 93.0%. When compared at equivalent mass concentrations, RFL-DW20-EP exhibited stronger NO-inducing activity than crude RFL-DW20, whereas RFL-DW20-ES showed little or no NO-inducing activity (Fig. 7B). Notably, the greater activity of RFL-DW20-EP was not accompanied by enrichment of total carbohydrates, polyphenols, or flavonoids. Conversely, RFL-DW20-ES contained relatively high levels of these constituents despite showing little or no NO-inducing activity. These results indicate that the enhanced macrophage-stimulating activity of RFL-DW20-EP cannot be explained simply by enrichment of total carbohydrates, polyphenols, or flavonoids and suggest that specific ethanol-precipitable constituents may be responsible for the observed activity.

### HPLC Chromatographic Fingerprinting of RFL-DW20 and Its Ethanol Fractions

**Fig. 8:**
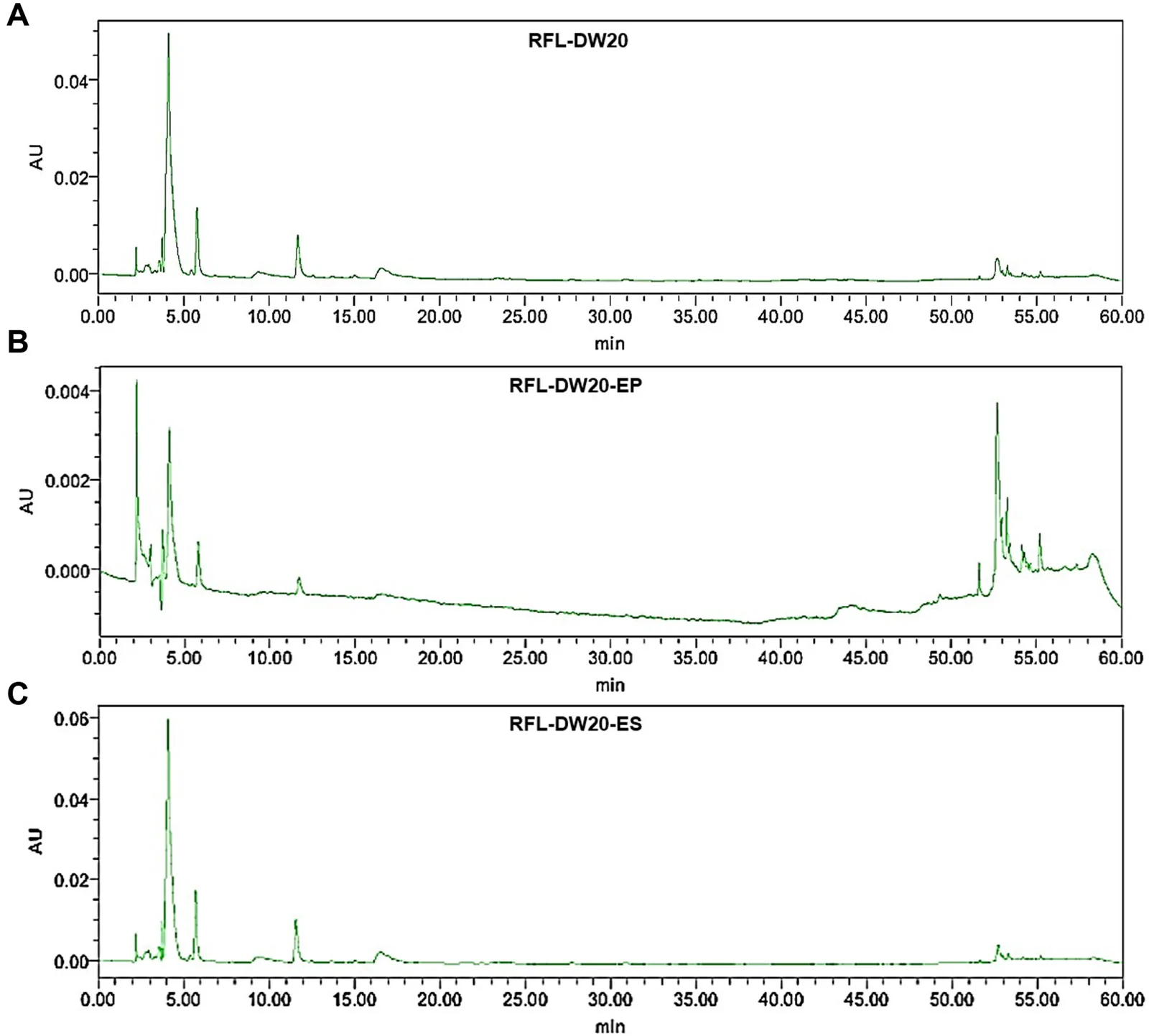
HPLC chromatographic fingerprints of RFL-DW20 and its ethanol fractions. HPLC-PDA chromatograms of (**A**) RFL-DW20, (**B**) RFL-DW20-EP, and (**C**) RFL-DW20-ES were recorded at 254 nm. Chromatographic separation was performed on a SunFire C18 column (5 μm, 100 Å, 250 × 4.6 mm) using water and acetonitrile as the mobile phases with gradient elution. The flow rate was 1.0 mL/min, the injection volume was 10 μL, and the column temperature was maintained at 25°C. The y-axis scales were independently adjusted to facilitate visualization of the chromatographic profiles.

To further characterize the chemical profiles of RFL-DW20 and its ethanol fractions, HPLC-PDA chromatographic fingerprint analysis was performed at 254 nm. RFL-DW20 exhibited several prominent UV-detectable peaks, predominantly in the early region of the chromatogram (Fig. 8A). RFL-DW20-ES showed several major chromatographic features similar to those observed in the crude RFL-DW20 (Fig. 8C). In contrast, RFL-DW20-EP exhibited substantially lower overall absorbance at 254 nm and a chromatographic pattern distinct from those of RFL-DW20 and RFL-DW20-ES (Fig. 8B). Notably, the distribution of the major UV-detectable constituents did not parallel the macrophage-stimulating activity observed after ethanol fractionation. Although RFL-DW20-EP exhibited a less intense chromatographic profile at 254 nm, it showed greater NO-inducing activity than crude RFL-DW20, whereas RFL-DW20-ES retained several prominent chromatographic features but showed little or no NO-inducing activity (Fig. 7B). These findings demonstrate that ethanol precipitation produces a chemically distinguishable fraction and suggest that the enrichment of macrophage-stimulating activity in RFL-DW20-EP is not associated with the major constituents detected at 254 nm.

## Discussion

The present study demonstrates that the water extract of *R. fasciculatum* leaves prepared at 20°C for 24 h (RFL-DW20) activates macrophage immune responses in RAW264.7 cells through a TLR4-dependent JNK and NF-κB signaling pathway. RFL-DW20 increased the production of NO, PGE2, IL-6, and TNF-α, upregulated the expression of iNOS and COX-2, and enhanced neutral red uptake without causing cytotoxicity. Mechanistically, the macrophage-activating effects of RFL-DW20 were strongly suppressed by TAK-242, SP600125, and BAY 11-7082, indicating the functional involvement of TLR4, JNK, and NF-κB. In addition, RFL-DW20 induced rapid phosphorylation of JNK and NF-κB p65, and these phosphorylation events were blocked by TAK-242. These findings support the conclusion that RFL-DW20 promotes macrophage activation by triggering TLR4-mediated activation of JNK and NF-κB signaling.

Macrophages are essential effector cells of innate immunity that participate in phagocytosis, antigen presentation, cytokine secretion, and coordination of host defense responses [5, 6]. Because activated macrophages produce various immune mediators that contribute to the elimination of invading pathogens and communication with other immune cells, the appropriate activation of macrophages has been regarded as an important cellular basis for immune enhancement [6]. In the present study, RFL-DW20 increased NO production together with iNOS mRNA and protein expression. NO produced by iNOS is a major macrophage-derived effector molecule involved in antimicrobial and cytotoxic defense mechanisms [7]. RFL-DW20 also enhanced PGE2 production and COX-2 expression. Although PGE2 is often discussed in the context of inflammation, it also contributes to the regulation of immune responses depending on the cellular context, receptor subtype, and local microenvironment [22]. Therefore, the simultaneous induction of NO, PGE2, iNOS, and COX-2 by RFL-DW20 indicates that this extract activates macrophages at both functional and molecular levels.

The ability of RFL-DW20 to induce IL-6 and TNF-α further supports its macrophage-stimulating activity. Macrophage-derived cytokines play important roles in innate immune communication and in shaping subsequent adaptive immune responses [8]. In this study, RFL-DW20 concentration-dependently increased the mRNA expression of IL-6 and TNF-α. However, the secretion patterns of these cytokines were not identical: IL-6 secretion increased in a concentration-dependent manner, whereas TNF-α secretion was elevated but remained relatively similar across the tested concentrations. This discrepancy between transcriptional induction and secreted protein levels may reflect cytokine-specific regulatory mechanisms, including differences in mRNA stability, translational efficiency, secretion kinetics, or saturation of TNF-α release within the tested concentration range. Notably, although RFL-DW20 markedly increased IL-1β mRNA expression, extracellular IL-1β levels were not significantly altered. This dissociation between IL-1β transcription and extracellular accumulation indicates that transcriptional induction alone was not sufficient to produce a corresponding increase in extracellular IL-1β under the present experimental conditions. Because IL-1β release involves additional post-transcriptional processing and secretion mechanisms, the observed response may reflect cytokine-specific regulation at steps downstream of transcription. However, because IL-1β processing and inflammasome-associated signaling were not directly examined in the present study, the mechanism underlying this discrepancy remains to be determined. Taken together, these findings indicate that RFL-DW20 induces distinct transcriptional and secretory responses among macrophage-associated cytokines rather than uniformly increasing all pro-inflammatory cytokines.

Importantly, RFL-DW20 also increased neutral red uptake, indicating that the extract not only induced soluble immune mediators but also enhanced macrophage functional activity. Since phagocytosis is one of the most representative functions of macrophages in innate host defense [6], this finding strengthens the biological relevance of RFL-DW20-mediated macrophage activation.

A notable finding of this study is that the macrophage-activating activity of *R. fasciculatum* leaves was strongly dependent on extraction conditions. Among water, 30% ethanol, 50% ethanol, and 70% ethanol extracts, only the water extract induced NO production. Furthermore, among water extracts prepared at different temperatures, only the extract prepared at 20°C showed NO-inducing activity, whereas extracts prepared at 40°C, 60°C, or 80°C were inactive. These results suggest that the active constituents responsible for macrophage activation are preferentially recovered under mild aqueous extraction conditions and may be unstable or structurally altered by elevated temperature during extraction. The expanded extraction-time analysis revealed that extracts prepared for up to 12 h showed little or no NO-inducing activity, whereas the activity became evident at 16 h and progressively increased through 20 and 24 h. No further increase was observed when the extraction period was extended to 48 h, indicating that macrophage-stimulating activity reached a plateau by 24 h. The delayed increase in activity may reflect the gradual extraction or solubilization of specific water-soluble constituents associated with macrophage activation. However, because the active constituents have not yet been identified, the chemical basis underlying this extraction-time-dependent profile remains to be elucidated. The loss of activity after autoclaving RFL-DW20 in aqueous solution, but not in powder form, further supports the possibility that the active components may be sensitive to heat-induced alteration in hydrated conditions. This property distinguishes RFL-DW20 from LPS, which retained NO-inducing activity after autoclaving.

Because endotoxin contamination is a major concern in studies of macrophage activation, especially when testing natural extracts or high-molecular-weight fractions [23], we employed complementary approaches to address this issue. PMB strongly suppressed LPS-induced NO production but did not reduce RFL-DW20-induced NO production. In addition, autoclaved LPS retained its activity, whereas RFL-DW20 showed a different heat-response pattern. Importantly, direct endotoxin quantification showed that the endotoxin level in RFL-DW20 at 100 μg/mL was below 0.01 EU/mL, with fluorescence signals comparable to those of the 0 EU/mL standard and endotoxin-free water (Supplementary Fig. S1 and Table S1). Taken together, these findings provide additional evidence that the macrophage-activating activity of RFL-DW20 is unlikely to result from endotoxin contamination. This point is particularly important because several plant- or microbe-derived macromolecular preparations can activate macrophages, and endotoxin exclusion is necessary for reliable interpretation of immunostimulatory activity [24, 25].

The receptor-screening experiments identified TLR4 as the major receptor involved in RFL-DW20-mediated macrophage activation. TAK-242 markedly inhibited RFL-DW20-induced NO and PGE2 production, iNOS and COX-2 mRNA expression, IL-6 and TNF-α expression and secretion, and neutral red uptake. In contrast, C29 and mannan only weakly reduced NO production, and laminarin had little effect. These results suggest that TLR4 is the dominant receptor for RFL-DW20-induced macrophage activation, although minor involvement of other receptors, such as TLR2 or mannose receptor-associated pathways, cannot be completely excluded. TLR4 is a major pattern-recognition receptor that initiates innate immune signaling and activates downstream transcription factors, including NF-κB, as well as MAPK pathways [11, 26]. Therefore, the strong inhibitory effect of TAK-242 is consistent with the established role of TLR4 in macrophage activation.

Downstream signaling analysis revealed that RFL-DW20 activated ERK1/2, p38, JNK, and NF-κB p65, demonstrating that macrophage stimulation by RFL-DW20 engages multiple signaling pathways. Importantly, however, activation of a signaling pathway did not necessarily indicate its functional requirement for NO production. Although RFL-DW20 induced phosphorylation of ERK1/2 and p38, pharmacological inhibition with PD98059 and SB203580, respectively, did not significantly affect RFL-DW20-induced NO production. These findings indicate that ERK1/2 and p38 are activated in response to RFL-DW20 but are dispensable for NO production under the present experimental conditions. In contrast, inhibition of JNK by SP600125 and NF-κB by BAY 11-7082 markedly suppressed RFL-DW20-induced NO production and also reduced PGE2, IL-6, TNF-α, and neutral red uptake, supporting major functional roles for JNK and NF-κB in RFL-DW20-mediated macrophage activation. Western blot analysis further showed rapid phosphorylation of JNK and NF-κB p65. Moreover, RFL-DW20 induced rapid IκBα phosphorylation followed by a marked reduction in total IκBα, providing additional biochemical evidence supporting activation of the canonical NF-κB pathway. TAK-242 markedly attenuated RFL-DW20-induced phosphorylation of JNK and p65, further placing TLR4 upstream of these signaling events. Collectively, these findings support TLR4-dependent JNK and canonical NF-κB signaling as major pathways contributing to RFL-DW20-mediated macrophage activation, while ERK1/2 and p38 appear to be activated but dispensable for the NO response.

The chemical profiling and ethanol fractionation results provide additional insight into the constituents associated with the macrophage-stimulating activity of RFL-DW20. RFL-DW20-EP represented 23.0% of the crude extract mass, whereas RFL-DW20-ES accounted for 70.0%. Despite its relatively low recovery yield, RFL-DW20-EP exhibited greater NO-inducing activity than crude RFL-DW20 when compared at equivalent mass concentrations, indicating enrichment of macrophage-stimulating activity on a mass basis in the ethanol-precipitated fraction. Interestingly, this enrichment of biological activity was not accompanied by enrichment of total carbohydrates, polyphenols, or flavonoids. RFL-DW20-EP contained substantially lower levels of these constituents than crude RFL-DW20, whereas the relatively inactive RFL-DW20-ES contained carbohydrate, polyphenol, and flavonoid levels comparable to or higher than those of the crude extract. Therefore, the enhanced macrophage-stimulating activity of RFL-DW20-EP cannot be explained simply by the total amounts of these chemical classes. HPLC-PDA fingerprint analysis further demonstrated that ethanol fractionation markedly altered the chemical profile of RFL-DW20. RFL-DW20-ES retained several of the major UV-detectable chromatographic features observed in the crude extract, whereas RFL-DW20-EP exhibited substantially lower overall absorbance and a distinct chromatographic pattern at 254 nm. Notably, this chromatographic distribution did not parallel the macrophage-stimulating activity, which was enriched in RFL-DW20-EP rather than in RFL-DW20-ES. These findings indicate that neither the bulk amounts of carbohydrates, polyphenols, and flavonoids nor the major constituents detected at 254 nm readily account for the enriched macrophage-stimulating activity of the EP fraction. However, because HPLC-PDA fingerprinting at 254 nm detects only constituents possessing suitable UV-absorbing chromophores, the present chromatographic analysis does not provide comprehensive structural characterization of RFL-DW20-EP. Accordingly, the active constituent(s) cannot currently be assigned to a specific chemical class, and further purification and structural characterization will be required to determine their chemical identities. Previous studies on *R. fasciculatum* have mainly focused on anti-obesity [19, 20], antioxidant [21], anti-aging [21], and allergy-relief effects [18]. In particular, Jung *et al*. (2014) [18] showed that *R. fasciculatum* attenuated allergic inflammation and reduced inflammatory mediator production. At first glance, these reports may appear different from the present findings, in which RFL-DW20 stimulated macrophage mediator production in unstimulated RAW264.7 cells. However, this difference is likely attributable to differences in extraction solvent, plant part, fraction composition, experimental model, and immune status of the cells. Anti-inflammatory effects in overactivated macrophages and immunostimulatory effects in resting macrophages are not necessarily contradictory; rather, they may reflect context-dependent immunomodulatory potential of distinct constituents from the same plant species. The present study is distinct from previous reports because it identifies a low-temperature water extract of *R. fasciculatum* leaves as a macrophage-activating material and provides mechanistic evidence for TLR4-dependent JNK/NF-κB signaling.

## Conclusion

In conclusion, this study demonstrates that RFL-DW20 enhances macrophage immune responses by increasing NO, PGE2, IL-6, and TNF-α production, inducing iNOS and COX-2 expressions, and promoting neutral red uptake in RAW264.7 cells. These effects are mediated predominantly through TLR4-dependent activation of JNK and NF-κB signaling pathways. In addition, the NO-inducing activity of RFL-DW20 was enriched in the ethanol-precipitated fraction, which exhibited a chromatographic profile distinct from those of the crude extract and ethanol-soluble fraction. These findings indicate that the biologically active ethanol-precipitated fraction is chemically distinguishable from the crude extract and ethanol-soluble fraction, although the constituent(s) responsible for the macrophage-stimulating activity remain unidentified. These findings provide new evidence that *R. fasciculatum* leaf water extract may be developed as a natural immune-stimulating functional material. Nevertheless, this study has several limitations. First, the immunostimulatory activity of RFL-DW20 was evaluated only in RAW264.7 macrophages, and its effects need to be validated in primary macrophages and in vivo models. Second, the active constituents in RFL-DW20-EP were not structurally identified. Finally, genetic approaches targeting TLR4, JNK, and NF-κB would be useful to further confirm the pharmacological inhibitor-based mechanism proposed in this study.

## Supplemental Materials

Supplementary data for this paper are available on-line only at http://jmb.or.kr.
